# Is leisure time sitting associated with mortality rates among men diagnosed with localized prostate cancer?

**DOI:** 10.1097/CEJ.0000000000000523

**Published:** 2019-07-23

**Authors:** Stephanie E. Bonn, Erik Holmberg, Jonas Hugosson, Katarina Bälter

**Affiliations:** a Clinical Epidemiology Division, Department of Medicine (Solna); b Department of Medical Epidemiology and Biostatistics, Karolinska Institutet, Stockholm, Sweden; c Department of Oncology; d Department of Urology, Institute of Clinical Sciences, Sahlgrenska Academy at University of Gothenburg; e Regional Cancer Center Western Sweden, Gothenburg; f School of Health, Care and Social Welfare, Division of Public Health Sciences, Mälardalen University, Västerås, Sweden

**Keywords:** adults, cohort, physical activity, prostate cancer, sitting time, survival

## Abstract

**Objective:**

Being physically active postdiagnosis has been associated with lower rates of prostate cancer progression and mortality, but studies investigating postdiagnostic time spent sitting are lacking. We aim to study the association between leisure time sitting after a prostate cancer diagnosis and overall and prostate cancer-specific mortality.

**Methods:**

Data from 4595 men in Sweden, diagnosed with localized prostate cancer between 1997–2002 and followed-up until the end of 2012, were analyzed. Time spent sitting during leisure time postdiagnosis was categorized into <2, 2–3, 3–4, and >4 h/day. Multivariable-adjusted Cox proportional hazards models were used to estimate hazard ratios (HRs) with 95% confidence intervals (CI) of postdiagnosis leisure time sitting and a joint variable of sitting time and exercise, and time to overall or prostate cancer-specific death.

**Results:**

The results showed no significant associations between postdiagnostic leisure time sitting and overall or prostate cancer-specific mortality rates. When the joint effect of both sitting and exercise time was considered, borderline significantly lower mortality rates for overall and prostate cancer-specific mortality were seen among participants that sat the least and exercised the most compared to the reference category with participants sitting the most and exercising least (HR: 0.75; 95% CI: 0.56–1.00 and HR: 0.61; 95% CI: 0.36–1.05, respectively).

**Conclusions:**

No significant association between leisure time sitting and mortality rates among men diagnosed with localized prostate cancer was seen. This study does not support an association between leisure time sitting *per se*; however, being physically active may have beneficial effects on survival among men diagnosed with localized prostate cancer.

## Introduction

Prostate cancer is the most common cancer among men in the Western world (Torre *et al.*, 2016). Over 90% of all men diagnosed with prostate cancer in the US are diagnosed with localized disease for which survival rates are high, meaning that the number of men ever diagnosed with prostate cancer is growing (Miller *et al.*, 2016). Identifying lifestyle factors that influence survival is therefore important.

Physical activity and sedentary time (i.e. waking time spent on an activity intensity level ≤1.5 METs, metabolic equivalents, while in a sitting, lying or reclining posture) (Tremblay *et al.*, 2017) are two separate, modifiable behaviors. While the positive effects of physical activity in relation to chronic disease and mortality are well studied, sedentary behavior is still an emerging field of research. Sedentary time has, nevertheless, been recognized as a risk factor for several chronic diseases independent of time spent in moderate-to-vigorous physical activity (MVPA) (Dunstan *et al.*, 2012), including cancer mortality in the general population (Keadle *et al.*, 2015).

While increased postdiagnosis physical activity has been associated with lower rates of both cancer progression (Richman *et al.*, 2011), and all-cause (Bonn *et al.*, 2015; Friedenreich *et al.*, 2016) as well as prostate-specific mortality (Kenfield *et al.*, 2011; Bonn *et al.*, 2015; Friedenreich *et al.*, 2016), evidence of effects of postdiagnostic time spent sedentary and survival among patients is still lacking. To our knowledge, only one study by Friedenreich *et al.* (2016) has, to date, looked at sedentary behavior postdiagnosis and mortality among men diagnosed with prostate cancer. More specifically, this study only examined occupational sedentary time, and studies investigating sedentary time in different domains of life are lacking.

We aim to investigate the effect of leisure time sitting after a prostate cancer diagnosis on overall and prostate cancer-specific mortality in a cohort of 4623 Swedish men diagnosed with localized disease.

## Methods

### Study design

The PROCAP (Progression in Cancer of the Prostate) study has been described previously (Szulkin *et al.*, 2012). Briefly, study participants were derived from a retrospective nationwide cohort study of men with localized prostate cancer, the National Prostate Cancer Register (NPCR) of Sweden Follow-up Study (Stattin *et al.*, 2008). Men with a registered localized prostate cancer in the Swedish NPCR between 1 January 1997 (1 January 1998, in one region) and 31 December 2002 were eligible for participation in the NPCR of Sweden Follow-up Study. The NPCR (Van Hemelrijck *et al.*, 2013) includes 98% of the prostate cancer cases registered in the Swedish National Cancer Registry (NCR) (Barlow *et al.*, 2009), which holds information on all incident cancers in Sweden. The NPCR contains additional information including serum prostate-specific antigen (PSA) levels, tumor–node–metastasis (TNM) stage, tumor differentiation at time of diagnosis and primary treatment. Additional inclusion criteria were diagnostic serum PSA < 20 ng/mL, local tumor stage T1-T2, no signs of lymph node metastasis (NX or N0), or bone metastasis (MX or M0), and being ≤70 years of age at diagnosis. Of the 8304 who fulfilled the criteria, 7960 (96%) accepted inclusion to the study. Men in the NPCR of Sweden Follow-up Study who were still alive in 2007 (n = 7075) were eligible for and invited to participate in the PROCAP study.

In total, 5779 (82%) men agreed to participate in PROCAP. Participants responded to a questionnaire assessing lifestyle factors either on the web (50%) or in paper format (50%) and donated a blood sample for genetic analysis between January 2007 and June 2008. For analyses in the present study, men with missing clinical information (n = 290) or treated with hormone therapy (n = 118) were excluded. Additionally, men who did not complete the questionnaire (n = 341) or were missing information on leisure time sitting (n = 435) were also excluded. The final analytical sample for leisure time sitting comprised 4595 men.

Prostate cancer-specific and all-cause mortality were end points in the present study. Date and cause of death were obtained by linkage to the Swedish Cause-of-Death Registry. Time from prostate cancer diagnosis to date of death or censoring on 31 December 2012, whichever came first, was used as the underlying time scale. The study was approved by the research ethics board at Karolinska Institutet (Stockholm, Sweden) and all patients included in PROCAP gave their written informed consent for participation at the time of inclusion.

### Exposure assessment

Time spent sitting during leisure time after prostate cancer diagnosis was assessed using a previously validated question (Norman *et al.*, 2001). Participants were asked to report the time they spent sitting, reading or watching television during leisure time ‘after diagnosis’ and were given seven predefined response alternatives: <1 h/day, 1–2 h/day, 2–3 h/day, 3–4 h/day, 4–5 h/day, 5–6 h/day and >6 h/day. Few participants reported leisure time sitting in the lowest category of <1 h/day (n = 261, 5.7%), or in the highest categories of 5–6 h/day (n = 138, 3.0%) or >6 h/day (n = 120, 2.6%). To ensure a sufficient number of cases in each category, the first two response alternatives were combined into <2 h/day and the three highest categories of 4–5 h/day, 5–6 h/day and >6 h/day were combined into >4 h/day. Final analysis of leisure time sitting was made using categories of <2 h/day, 2–3 h/day, 3–4 h/day and >4 h/day.

### Assessment of covariates

In addition to clinical variables, that is, primary treatment, serum PSA-level, TNM-stage and Gleason score, that were obtained from NPCR, several additional covariates were examined as potential confounding factors. Self-reported smoking habits after diagnosis (current, former or never smoker), education level (≤9, 9 to ≤12, or >12 years), employment status during the past year (retired/working) and having a relative with prostate cancer (yes/no) were assessed in the lifestyle questionnaire at inclusion in PROCAP. BMI (BMI: <25, 25 to <30, or ≥30 kg/m^2^) at diagnosis was calculated from self-reported current height and weight at the time of responding to the questionnaire adjusted for reported weight change since diagnosis. An additional categorical variable of weight change was also created categorizing patients into three groups: no change or a change ≤5%, an increase >5%, or a decrease >5% since diagnosis. Information on physical activity after diagnosis was obtained from a questions assessing exercise during leisure time (<1 vs. ≥1 h/week) (Bonn *et al.*, 2015).

### Statistical analysis

Characteristics of study participants are presented as means with SD or distributions (n, %) and differences between men in categories of leisure time sitting were tested using one-way ANOVA for continuous variables and *χ*^*2*^ test for categorical variables. Overall and prostate cancer-specific mortality were analyzed using the Kaplan–Meier method. Time to event for the different categories of leisure time sitting was compared using the log-rank test.

To estimate unadjusted, age-adjusted and multivariable-adjusted hazard ratios (HRs) and 95% confidence intervals (CI), Cox proportional hazards models using time since prostate cancer diagnosis as the underlying time scale were used. By study design, all patients were left truncated at the date of inclusion to PROCAP. Leisure time sitting was included as a categorical variable in the models with the lowest level (<2 h/day) used as the reference. The Cox proportional hazards assumption was tested using Schoenfelds residual.

Potential confounding factors to adjust for in the Cox proportional hazards models were tested for statistical associations to the exposure using one-way ANOVA for continuous variables and *χ*^*2*^ test for categorical variables, and to the mortality outcomes in Cox proportional hazards models. Based on this confounder-selection and subject matter knowledge, results from two multivariable adjusted models are presented. The first model adjusts for age at diagnosis, BMI, primary treatment, employment status and leisure time exercise while the second model additionally adjusts for clinical variables of serum PSA, TNM-stage and Gleason score at prostate cancer diagnosis. Variables tested that were not included in final models were as follows: weight change after diagnosis, smoking habits, level of education and family history of prostate cancer. Sensitivity analysis using a lag-time of 18-months (excluding men who died within 18 months of responding to the questionnaire) were performed to examine whether the reported level of leisure time sitting was influenced by illness.

To investigate a potential joint association between sitting time and exercise, a combined variable of these behaviors was created. Participants were divided into four categories: (1) sitting time ≥3 h/day + exercise <1 h/day, (2) sitting time ≥3 h/day + exercise ≥1 h/day, (3) sitting time <3 h/day + exercise <1 h/day and (4) sitting time <3 h/day + exercise ≥1 h/day. HRs and 95% CIs were obtained from Cox proportional hazards models with the least active category (sitting time ≥3 h/day + exercise <1 h/day) used as a reference. Additional sensitivity analyses with 18-months lag-time were also performed.

All analyses were performed using STATA 14.0 (STATA Corporation, College Station, TX). The level of significance was set to α = 0.05.

## Results

Among the 4595 men included in final analysis, 1352 reported <2 h/day of leisure time sitting, 1551 reported 2<3 h/day, 1023 reported 3<4 h/day and 669 men reported sitting ≥4 h/day during their leisure time. Characteristics of the study participants are shown in Table 1. Differences in characteristics were seen between sitting time groups with regards to age, exercise, education, weight change/stability, primary treatment and family history of prostate cancer. The average follow-up time for all participants was 12.1 (1.7) years. In total, 537 deaths from any cause occurred of which 169 (31.5%) were prostate cancer specific. In the lag-time analysis, 119 men who died within 18-months after responding to the questionnaire were excluded.

**Table 1:**
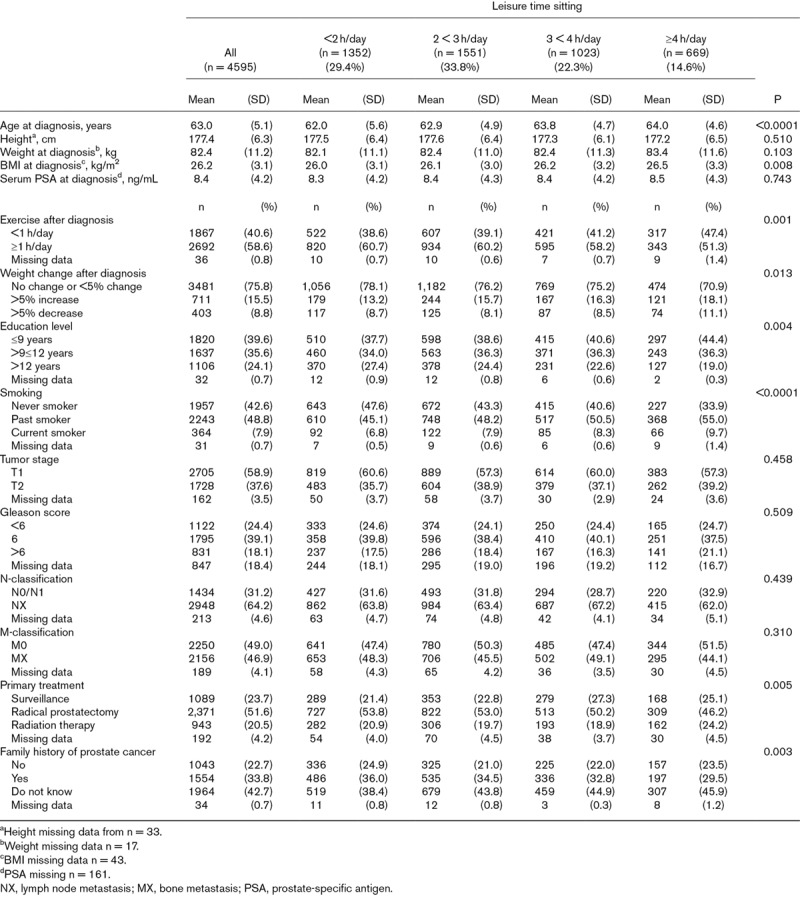
Characteristics of study participants included in analysis in the PROCAP-study according to daily time spent sitting during leisure time

**Fig. 1:**
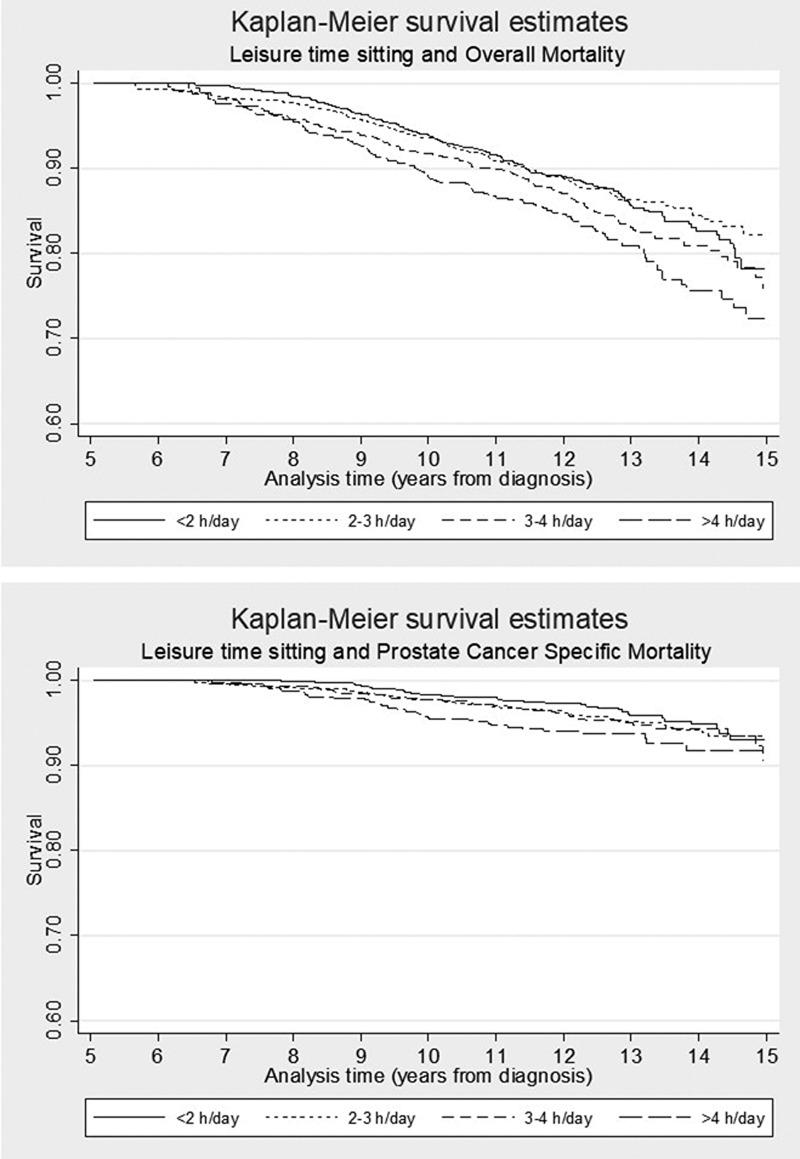
Kaplan–Meier survival curves for overall (top) and prostate cancer specific (bottom) mortality by categories of leisure time sitting. The x-axis shows time from inclusion in PROCAP (Progression in Cancer of the Prostate) to death or censoring, origin (time = 0) is date of prostate cancer diagnosis in left-truncated Cox proportional hazards regression models.

Results from analysis of sitting time from crude, age-adjusted and multivariable-adjusted Cox proportional hazards models are shown in Table 2. For overall mortality, Kaplan–Meier curves (Fig. 1a) with a log-rank test (*P* = 0.008) indicate an increased mortality rate among men reporting the highest amount of sitting during leisure time (≥4 h/day). However, no statistically significant differences in mortality rates were seen between the categories of sitting during leisure time in multivariable-adjusted models in main or sensitivity analysis. For prostate cancer-specific mortality, Kaplan–Meier curves (Fig. 1b) also indicate a higher mortality rate among men in the highest category of sitting time, although the log-rank test was not statistically significant (*P* = 0.18). Nevertheless, although not statistically significant in multivariable adjusted models, our crude models indicate potentially increased overall (HR: 1.34, 95% CI: 1.04–1.72) and prostate cancer-specific (HR: 1.64, 95% CI: 1.04–2.57) mortality rate among men reporting sitting ≥4 h/day compared to men reporting sitting <2 h/day.

**Table 2:**
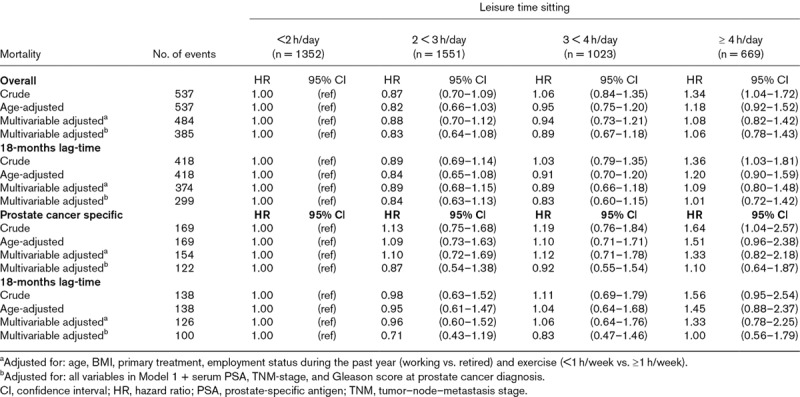
Survival analysis of time spent sitting during leisure time and overall and prostate cancer-specific mortality, HRs with 95% CIs

**Fig. 2:**
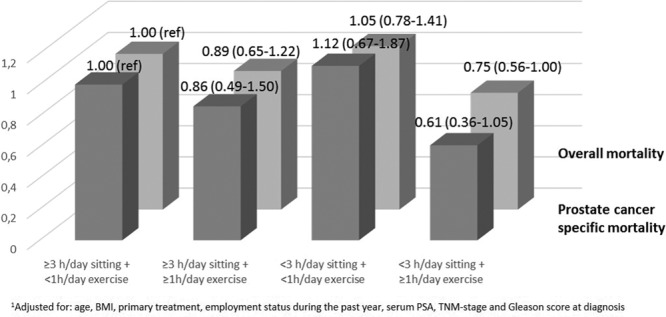
Survival analysis of the joint effect between sitting time and physical activity and overall and prostate cancer-specific mortality, HRs with 95% CIs from multivariable adjusted models^1^ [adjusted for age, BMI, primary treatment, employment status during the past year (working vs. retired), serum PSA, TNM-stage and Gleason score at prostate cancer diagnosis]. CI, confidence interval; HR, hazard ratio; PSA, prostate-specific antigen; TNM, tumor–node–metastasis stage.

Thereafter, we explored the joint effect of both sitting and exercise time and results from multivariable adjusted models are presented in Fig. 2. For overall and prostate cancer mortality, a borderline statistically significantly lower mortality rate was seen among participants who belonged to the combined group of short sitting time (<3 h/day) and long exercise time (≥1 h/day), compared to the reference group with participants with long sitting time (≥3 h/day) and the short exercise time (<1 h/day) (HR: 0.75; 95% CI: 0.56–1.00 and HR: 0.61; 95% CI: 0.36–1.05, respectively). Although not statistically significant, the HR for participants in the high sitting and high exercise time indicated lower overall and prostate cancer-specific mortality rates compared to the reference. No association was seen for those in the short sitting and exercise time compared to the reference category. Results remained similar in lag-time sensitivity analysis.

## Discussion

In this large cohort of men diagnosed with localized prostate cancer, we did not find any statistically significant associations between self-reported time spent sitting during leisure time after diagnosis and overall or prostate cancer-specific mortality rates.

Analysis of the joint effect of sitting and exercise time showed that exercise seemed to have a large impact on mortality rates, and men who reported to sit the least and exercise the most had the largest decrease in both overall and prostate cancer-specific mortality rates. We have previously reported reduced mortality rates among more physically active men compared to less active men in the same cohort (Bonn *et al.*, 2015). Additionally, Kenfield *et al.* (2011) have shown a significant inverse association between vigorous activity and prostate cancer-specific mortality and recently, Friedenreich *et al.* (2016) reported lower mortality rates among men with higher levels of postdiagnosis recreational physical activity compared to less active men. However, the association between sedentary time and mortality among prostate cancer patients remains less clear.

Self-reported time spent watching television has, independent of time spent in MVPA, been associated with increased cancer mortality rates in the general population (Keadle *et al.*, 2015). Friedenreich *et al.* (2016) studied postdiagnosis occupational sedentary behavior among men diagnosed with prostate cancer and, in line with our findings, saw no statistically significant trend between sedentary time and mortality. Important to acknowledge is that while Friedenreich *et al.* (2016) investigated occupational sedentary time, the present study assessed sitting during leisure time. However, the effect of sedentary time accumulated within different domains of life, for example, occupational, leisure time or during transportation, may not differ and future studies looking at total sedentary time are also needed. Further, the vast majority of men in the present study were retired during follow-up as the mean age at diagnosis was 63 years; as such, occupational sitting time was not assessed specifically. The association between sedentary time and risk of incident prostate cancer is also unclear and a recent study showed no association with total sitting time or time spent watching television (Lynch *et al.*, 2014).

Noteworthy strengths of the present study include the population based design, large sample size and long follow-up time. A limitation to the study design is that only men who were still alive 5–10 years after their prostate cancer diagnosis were included. Results are therefore conditioned on having survived long enough. However, survival rates among men diagnosed with localized prostate cancer is high with a 15-year survival >90% (Miller *et al.*, 2016). The left-truncation of data is therefore likely to result in conservative estimates. Another limitation is the potential for reverse causation. Additional sensitivity analyses using an 18-month lag-time were performed to account for this. Since results remained similar, we do not believe that our results are an artefact of reverse causality. Additionally, this study used a self-reported measure of sedentary time and potential misclassification and measurement error biasing results towards a null effect cannot be ruled out. Nonetheless, the question about leisure time sitting used has been validated previously and showed a modest correlation (Spearman *r* = 0.51) with a physical activity record (Norman *et al.*, 2001).

Subjective methods including questionnaires to assess physical activity and sedentary behaviors have previously been standard in large epidemiological studies (Atkin *et al.*, 2012). However, sedentary time is often under reported in questionnaires, and objective assessment using for example accelerometers has been shown to provide more accurate measurement (Matthews *et al.*, 2018). During the past decade, accelerometer use has become feasible also in large epidemiological studies (Atkin *et al.*, 2012), although more validation studies of accelerometer use in older-adults are needed (Heesch *et al.*, 2018).

Future studies are needed to investigate different domains of sedentary time as well as total sedentary time and different sedentary behaviors, for example, accumulating sedentary time in longer bouts or breaking up sedentary time with short bouts of activity, using objective assessment methods, for example, accelerometers, in this patient group. Although the epidemiological evidence is still limited, physical activity may have beneficial effects on survival among men diagnosed with prostate cancer, but findings from our study and others do not support to date an association between leisure time sitting and mortality in this group.

## Acknowledgements

The authors thank Carin Cavalli-Björkman and Ami Rönnberg for their work during the data collection and Michael Broms for his work with the study data bases. We also acknowledge the hard work of all the research nurses who extracted data for the NPCR follow-up study.

This study was supported by grants from the Swedish Cancer Society (CAN 2011/868) and the Swedish Research Council for Health, Working life and Welfare (2011-0650).

## Conflicts of interest

There are no conflicts of interest.
